# Monitoring the Affordability of Healthy Eating: A Case Study of 10 Years of the Illawarra Healthy Food Basket

**DOI:** 10.3390/nu2111132

**Published:** 2010-11-19

**Authors:** Peter Williams

**Keywords:** food security, healthy food basket, food price

## Abstract

Healthy food baskets have been used around the world for a variety of purposes, including: examining the difference in cost between healthy and unhealthy food; mapping the availability of healthy foods in different locations; calculating the minimum cost of an adequate diet for social policy planning; developing educational material on low cost eating and examining trends on food costs over time. In Australia, the Illawarra Healthy Food Basket was developed in 2000 to monitor trends in the affordability of healthy food compared to average weekly wages and social welfare benefits for the unemployed. It consists of 57 items selected to meet the nutritional requirements of a reference family of five. Bi-annual costing from 2000–2009 has shown that the basket costs have increased by 38.4% in the 10-year period, but that affordability has remained relatively constant at around 30% of average household incomes.

## 1. Introduction

Food insecurity is strongly inversely associated with household and per capita income [1] and it has been estimated that the level of food insecurity as a consequence of limited resources is over 5% in the general Australian population [2]. A number of studies suggest that lower socio-economic families have diets that are less likely to comply with dietary guidelines [3,4], although this is not a consistent finding [5,6].

Differences in food prices between standard and healthier alternative products are thought to influence consumer choices, especially among the socioeconomically disadvantaged [7,8]. Several studies have concluded that a healthy diet can be more expensive unless significant changes are made to normal food patterns [9,10,11,12], and in general, when food selection is driven by cost considerations alone, resulting diets are energy-dense and nutrient poor [13].

For this reason many countries have undertaken regular surveys of the cost of healthy foods, as part of national nutrition monitoring and surveillance activities [14]. For over 100 years, the U.S. Department of Agriculture has prepared guides for selecting nutritious diets at different cost levels and their current Thrifty Food Plan is used by food assistance programs to determine the resources provided to low‑income households [15]. Canada officially standardized a national food basket in 1995 that is used to monitor the cost of an adequate diet [16]. It acts as a template for each province to adopt as a costing tool to reflect provincial differences in food availability, and many provinces update the costing annually [17,18].

### Issues influencing development of food baskets

In Australia a number of different food baskets have been developed for a variety of different purposes in each of the States: the Kimberley Market Basket in Western Australia [19]; the Northern Territory Nutritionists Market Basket Survey [20]; the Queensland Health Food Access Basket [21,22]; the Victorian Healthy Food Basket [23]; the Adelaide Healthy Food Basket in South Australia [24]; the New South Wales Healthy Food Basket [25]; and the Tasmanian Food Price Availability and Quality Survey [26]. This diversity of approaches has led to calls for the development of one common national approach [27].

However each of these baskets has slightly different objectives, which illustrates the diverse ways that food basket information can inform nutrition surveillance. Some of the aims can be to:

- Compare the price of healthy *versus* unhealthy food [28,29];
- Compare the price of healthy food in remote or rural *versus* metropolitan locations [19,30];
- Compare the availability of healthy foods in different geographic regions [31,32,3
- Compare food quality in different geographic regions [25,26];
- Calculate the minimum cost of an adequate diet for social policy planning [34];
- Develop educational material about low cost healthy eating [35];
- Calculate the environmental costs associated with different food patterns [36];
- Examine trends in food costs over time, including different food commodities [37,38];
- Monitor the changing affordability of a healthy diet compared to income and welfare support [24,39,40].

Given these different objectives, there have also been a variety of methods employed to define baskets of healthy foods. Some have used mathematical optimization models to define baskets of food that meet nutrition recommendations for minimum cost [41] while others have restricted the baskets to a few key food groups such as fruit and vegetables, or basic food staples [42,43]. Many have attempted to define food baskets based on objective nutritional criteria of what are healthy foods, excluding foods that would be regarded as unhealthy or indulgence foods [23,44]. Others have defined baskets based on data about normal food purchasing patterns, particularly when the focus of study is on food security [45]. Some baskets have attempted to combine recommendations about healthy eating with data on normal consumption, in order to make the baskets more realistic [46,47]. The family size for which the basket is constructed also varies. Baskets are usually designed to feed a family of between four and six people, but at least two recent baskets were defined for four different reference families, including single person households [23,46].

The Illawarra Healthy Food Basket (IHFB) was established in 2000. It consists of a basket of 57 foods, designed to meet the weekly nutritional requirements of a family of five in the Illawarra region (south of Sydney) in Australia. Results from surveys of the cost of the IHFB have been reported in full for the years 2000, 2001, 2003, 2005 and 2007 [48,49] and summary data from 2009 is available in a conference abstract [50].

The aim of the IHFB was to establish the basis for an ongoing survey of the affordability of a basket of healthy food items in one region of Australia, and to publish a regular index showing changes in the cost of the basket over time, compared to changes in average income levels and available social welfare benefits. This paper highlights some of the issues that have arisen in developing this healthy food basket, provides a summary of the findings from the six surveys conducted over a 10 year period, and reflects on the value and limitations of the use of food baskets for nutrition monitoring.

## 2. Methods

The methods used to define the foods included in the IHFB and the costing methods have been described in detail previously [48,51]. Briefly, the basket includes 10 breads and cereals, three dairy foods, 15 vegetables, six fruits, 10 meats, fish, poultry eggs and nuts, and 13 extra foods—including margarine, coffee, biscuits, ice-cream and vegemite. It was designed to conform to Australian dietary guidelines and to meet the targets of Australian recommended dietary intakes (RDIs) for a reference family of five (one 65-year old woman, two 39-year old parents, and two children—a 15-year old girl and a 5-year old boy). In each survey, the food items were priced in September at the same main supermarket, greengrocer and butcher in five suburbs of differing socio-economic status in the region, and the prices averaged across all the outlets, assuming half of all meat, fruit and vegetable items were purchased in the supermarkets. The basket cost was then compared with the average weekly earnings (AWE)—all employees’ total earnings in New South Wales—reported by the Australian Bureau of Statistics and the total welfare allowances available to the family assuming that no family member was employed.

## 3. Results and Discussion

### 3.1. Summary of finding from 10 years of surveys

Table 1 shows the cost of the IHFB over the 10 years of surveys, compared to the AWE and welfare benefits. The affordability of the basket, represented as a proportion of each of the two comparison weekly income sources, was relatively constant—ranging from 28 to 33% of family income, with no statistically significant trends over time. These results indicate that welfare payments and incomes have kept pace with the increase in the cost of the healthy food basket over this period. This does not appear to have been a specifically planned outcome, since indicative budget standards to calculate minimum costs of adequate standards of living are based mostly on movements in household expenditure surveys rather than the costs of healthy food baskets [52].

**Table 1:** The cost of the Illawarra Healthy Food Basket in Australian dollars compared with average weekly earnings * and welfare payments ** 2000–2009.

	2000	2001	2003	2005	2007	2009
**Weekly cost of IHFB ($)**	201.46	224.15	225.86	235.66	242.49	278.79
**AWE ($)**	675.10	706.50	772.70	836.10	865.10	923.40
**IHFB as % AWE**	29.8	31.9	29.2	28.2	28.0	30.2
**Total welfare payments per week ($)**	645.38	673.52	721.68	753.85	823.88	927.98
**IHFB as % welfare payments **	31.2	33.3	31.3	31.3	29.4	30.0

* For all employees, average total earnings in New South Wales in the May quarter;

** Welfare payments per week for the reference family (including aged pension, unemployment benefits, child support allowances and rental assistance).

These estimates of affordability are similar to estimates from other studies in Australia and overseas. In Australia, recent estimates of the costs of a healthy food basket for welfare-dependent families range from 31–40% of income [24,30,34,53]. Canadian estimates have also been around 30% [39]. Such estimates are typically higher than the measured levels of actual expenditure on food. By contrast, the 2003–2004 Household Expenditure Survey in Australia reported that Australian households in the lowest quintile of income spent only 21.1% of income on food and beverages [54].

**Figure 1:**
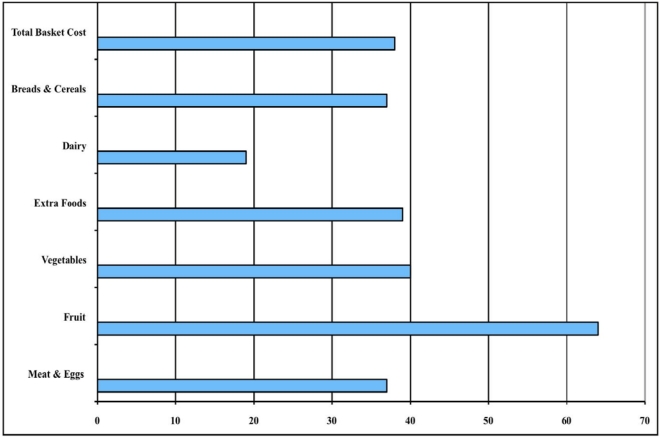
Percent change in the cost of the Illawarra Healthy Food Basket components 2000–2009.

The increase in the cost of the IHFB from 2000–2009 (38.4%) was proportional to the 37.6% rise experienced by the Consumer Price Index for food during the same period [55]. However, the cost increases were not uniform across all food categories. Figure 1 shows that the price increase for fruit (64%) was significantly higher than for all other foods (*p* < 0.05). This trend is of concern at a time when there has been a national campaign to increase the consumption of fruit and vegetables [56]. Increasing food costs might be a significant barrier to successful outcomes from health promotion activities, since it is known that consumers already perceive these foods to be expensive [57]. The reasons for the increases are multifactorial, including the impact of prolonged local droughts, increasing fuel and other production costs, and long-term climate changes affecting water available for irrigation. Policy approaches that focus on reducing costs (e.g., tax incentives for freight to remote locations) might therefore be more effective than consumer education on the health benefits of fruits and vegetables.

Several other results could be seen from the IHFB results. There was no consistent relationship between a suburb’s socioeconomic status and the basket prices, a finding that has been reported in other Australian surveys [24,42]. Furthermore, in most of the surveys the average price of the basket was lower if all fresh fruit and vegetables and meat were purchased from independent greengrocers and butchers rather than at the supermarkets. This finding is consistent with results in the United States as well [32] and could be useful in advice to consumers shopping on a limited budget.

### 3.2. Limitations and use of survey results

There are limitations with the IHFB surveys. The costing takes place in only one limited geographic region and it would be inappropriate to extrapolate results to other parts of Australia. Secondly, the sample of food outlets is limited and the surveys are undertaken at only one time point in the year and so may not reflect average costs over the whole year. Nonetheless, the consistency of the results with other surveys gives confidence in the usefulness of the trend data.

The discrepancies between the calculated percentage of income needed to buy the healthy basket and the typical proportion of household expenditure on food suggests that caution should be used in drawing conclusions about the absolute cost of healthy eating from food basket costing studies. There are several possible reasons for this. Firstly, food basket studies usually include mostly basic healthy food items that require preparation at home, and assume that all food will be consumed at home. Such food items may be more expensive than foods recorded in expenditure surveys, which include food purchased away from home as well those that may not be nutritionally ideal. For example, current fruit and vegetables intakes are well below recommended levels [58]. Secondly, the average household size in Australia is now only 2.0 persons, significantly lower than the reference families used in most basket studies, so the expenditure by these families is naturally less. Thirdly, the choice of food outlets surveyed, and the time of year (which can affect food prices) also influence results. Some studies use random samples of outlets, whereas others (like the IHFB) use a more limited convenience sample. Lastly, food baskets usually assume all food in the basket is eaten. By contrast, in Australia domestic food waste is estimated to be worth over $AUD11 per week per Australian household, and fruit and vegetables are the foods with the highest levels of waste [59].

Nonetheless, while the absolute estimates of affordability may not be reliable, results of trends over time are valuable, particularly for ongoing surveillance. Only two studies have been repeated regularly to provide this data: the Healthy Food Access Basket, which has been conducted five times since 1998 [22], and the IHFB. The former has measured costs and availability of healthy food in 78 stores across the state of Queensland, but the IHFB is the only longitudinal survey to measure affordability in comparison to income, and there are plans to continue the same survey on an ongoing basis. 

## 4. Conclusions

Food affordability, as measured by use of a healthy food basket, represents just one factor affecting food security. Methods of food production, the composition of the retail industry, social welfare policies, and cultural and technological changes all have impacts [14]. However, around the world, recent global economic and financial crises have resulted in higher food prices [43]. With increased warning that projected climate changes might put further significant upward pressure on food prices [60], it will be important to continue to undertake this type of monitoring into the future, to enable better targeting of activities to improve the diets of our populations.
